# Subjective assessment of occupational stress and mental health of nurses during the Covid-19 pandemic period

**DOI:** 10.3389/fpsyt.2023.1301113

**Published:** 2023-11-21

**Authors:** Krystyna Kowalczuk, Katarzyna Tomaszewska, Joanna Chilińska, Elżbieta Krajewska-Kułak, Marek Sobolewski, Justyna M. Hermanowicz

**Affiliations:** ^1^Department of Integrated Medical Care, Medical University of Bialystok, Bialystok, Poland; ^2^Department of Nursing, Institute of Health Protection, The Bronislaw Markiewicz State Higher School of Technology and Economics, Jarosław, Poland; ^3^Faculty of Health Science, Łomża State University of Applied Sciences, Łomża, Poland; ^4^Faculty of Management, Rzeszow University of Technology, Rzeszow, Poland; ^5^Department of Pharmacodynamics, Medical University of Bialystok, Bialystok, Poland

**Keywords:** stress, nurse, mental health, anxiety, depression, insomnia, work characteristics, COVID-19

## Abstract

**Introduction:**

Health status, sickness absence, and nurses’ attrition have a direct impact on the quality of care provided and patients’ health outcomes. The Covid-19 pandemic exacerbated issues that existed within the Polish healthcare system prior to the pandemic, including staff shortages, low wages, and system inadequacies. The aim of this study was to investigate how nurses during the Covid-19 pandemic period rated the burdensomeness of job characteristics and their mental health status, as well as the correlations between factors directly caused by the Covid-19 pandemic and nurses’ subjective assessments of job characteristics and mental health.

**Method:**

The cross-sectional study was conducted in January 2022, in Poland and involved 796 registered nurses working in hospitals.

**Results:**

Despite the pandemic’s sweeping societal effects, this research finds limited alteration in nurses’ perceptions of job stress and self-assessed mental health. Factors such as contact with infected patients, quarantine, and isolation do not appear to substantially modify mental health perceptions among nurses. Intriguingly, nurses subjected to COVID-19 testing report heightened stress and compromised mental health.

**Conclusion:**

The interplay of diverse factors influencing the well-being of nurses is intricately complex. It is advisable to prudently execute interventions and strategies to address the pandemic, aiming to alleviate its potential adverse effects on the mental health of nurses.

## 1 Introduction

Psychosocial determinants exert an adverse influence on employee well-being by inducing stress. These factors arise within distinct organizational and societal frameworks, their character molded by the subjective psychological appraisal of their relevance by the individual (1, 2). These factors can manifest as threats, constraints, value deprivations, or challenges to an individual’s abilities and aspirations (3). Psychosocial stimuli inherently display subjectivity. The potential for a specific aspect of a work environment to evolve into a psychosocial stressor is contingent upon the unique attributes of both the individual and the collective group within which the individual operates. The significance of a given factor for an employee hinges upon these contextual elements (4). Numerous studies reported that stressors occurring at work have a negative impact on the employee health (4–6). Psychosocial factors affect employees’ health by triggering a stress reaction that lasts for a long time (7). The effects of this reaction may be reflected in disorders of various systems and body functions. Therefore, it is not possible to establish a specific link between a specific psychosocial factor and the incidence of a specific disease (8).

The perception of workplace burdens is a subjective feeling, which has been confirmed by theories of occupational stress. The high social expectations and high professional demands placed on nurses by hospital managers and the relatively low salaries offered perfectly match the theory of the fit between the individual and the environment by French et al. (9), as well as Siegriest’s disequilibrium model (10).

French’s theory revolves around two fundamental components: the employee’s attitude level and their capacity to fulfill job requirements. Within this theory, a clear distinction is drawn between the objective reality of changes in the environment and the subjective perception thereof. Objective alignment relies on externally defined criteria, such as experience, education, and skills, which are typically evaluated by external experts, such as during a job interview. On the other hand, subjective alignment pertains exclusively to the individual attributes of the employee and their personal perception of the work environment. Mismatches can manifest in various patterns, each potentially influencing the level of stress experienced by the employee (9).

Siegirist’s model (10) illustrates the discrepancy between the effort invested in a job and the rewards obtained in return. The degree of effort is contingent upon two primary factors: the inherent demands of the job and the individual’s unique traits. Compensation for work encompasses three dimensions: financial remuneration and professional standing, recognition and support, and job stability and prospects for career advancement. When an employee becomes overly engrossed in their work, all the while undervaluing the rewards they receive, an imbalance between effort and reward emerges, resulting in a stressful situation (11).

We conducted the study on nurses, because it is nurses who are the most stressed group of health care workers, and one of the most stressed professional groups globally (12). Somatic and psychological stress-related illness indices are higher among nurses than in the general population (13, 14). Psychosocial burdens affect the development of anxiety, insomnia, excessive sleepiness and depression among nurses (15). The effects of loads manifest themselves as undesirable behaviors at work such as avoidance, increased irritability, cynical attitude. Any workload that occurs at work and exceeds the employee’s ability to cope with the workload is associated with absenteeism, change or resignation from work. Some nurses excessively use sick leave to avoid psychological strain at work (16–18). In Poland, a significant percentage of nurses leave the profession within 10 years of obtaining their professional qualifications. The main reasons for leaving the profession are low wages, difficult working conditions and poor health (19).

The work of a nurse is complex and involves multi-tasking. It requires a high level of manual dexterity for treatment and nursing activities, the meticulousness necessary for medical record keeping, the technical knowledge necessary for operating medical equipment and devices, interpersonal skills for collecting patient health history, patient health education and communication with their families. (20, 21).

Sources of stress experienced by nurses include poorly organized work, shift-based work that disrupts the natural biological rhythm of the body’s, irregular work that causes periodic high overloads, lack of satisfactory pay, lack of recognition from superiors, and lack of career development prospects. Nurses working in units with a significant risk of contact with chronic dying and death declare that the greatest sources of stress in their professional pacing include: dying and death of the patient, watching the development of the disease and its consequences, and the nurse–patient-family relationship (22). Direct contact with potentially infectious materials from the patient (blood, secretions, excretions) is an important source of stress (23). There is also often time pressure, as unpredictable situations arise, such as the sudden deterioration of a patient’s condition or resuscitation, wherein pure minutes are decisive for the life of the patient (24). There are also situations involving a sense of no control, such as when caring for an unconscious or intubated patient. At such time, the nurse has no opportunity to interact with the patient and get feedback on whether her work is having the intended effect. Many problems stem from nurses’ relationships with colleagues, patients and their families, which sometimes take place in an atmosphere of high emotional tension (22, 25).

When listing sources of stress at nursing work, it is important to emphasize the wide range of responsibilities associated with their professional duties, the pressure to be reliable and available, and the expectations of patients and their families (26). Society’s expectations of nurses are significantly different from feelings of nurses themselves. Societies expect nurses to express only those emotions that alleviate the fear and suffering of the patient and his family (27).

The COVID-19 pandemic has become an additional source of stress for health care workers including nurses what was confirmed in numerous studies (28–30). The pandemic brought a number of stressors to which health workers were exposed in the workplace. These included a high mortality rate for COVID-19 patients, more overtime shifts, fear of lack of appropriate medical equipment (including personal protective equipment), constant testing, quarantines, being in home isolation, contact with infected patients, infection by virus, risk of transmission to family members, fear of the viral spread at the workplace, lack of access to child care when working overtime or when schools were closed, as well as lack of support for other personal and family issues when faced with work demand increase (30, 31). Negative psychological factors were further exacerbated by media coverage of the pandemic, which focused on mortality among health care workers, and the disease in spread health care facilities (29).

As per the Centers for Disease Control and Prevention’s findings, healthcare professionals have undergone a decline in psychosocial work attributes, evidenced by deteriorating work environments, heightened irritability, reduced motivation, sensations of inadequacy, depressive symptoms, emotional overload, burnout, sleep disturbances, impaired interpersonal relationships, sensations of fatigue, feelings of being overburdened, and perceived threats (32).

We observed a paucity of research in the existing literature concerning the influence of the COVID-19 pandemic on the subjective perception of work characteristics and the mental well-being of Polish nurses. Consequently, we undertook an investigation to examine the potential associations between stressors unique to the COVID-19 crisis, namely, exposure to Sars-Cov-2-infected patients, extensive virus testing, quarantine, home isolation, and Sars-Cov-2 infection, and the subjective evaluation of psychosocial working conditions and the self-assessment of mental health among Polish nursing professionals.

## 2 Materials and methods

The cross-sectional study was executed in January 2022 within the Podlaskie Voivodeship in Poland. The participant cohort comprised 796 registered nurses employed at the Bialystok University Clinical Hospital and the Provincial Hospital in Lomza. The study protocols received endorsement from the Bioethical Commission of the Medical University of Bialystok, under reference no. APK.002.518.2021. All procedures undertaken during the investigation adhered to internationally recognized ethical principles governing research involving human subjects, encompassing aspects such as voluntary participation, informed consent, assurance of anonymity and confidentiality, mitigation of potential harm, and appropriate dissemination of results.

### 2.1 Study group selection

Owing to limitations in hospital accessibility in Poland during the study period, coupled with logistical challenges in arranging in-person encounters, the investigation was restricted to two hospitals located in Bialystok, the provincial capital, and Lomza, the second largest city within the province. The eligibility criteria for inclusion encompassed individuals employed within the hospital under a full-time contractual agreement. Nurses engaged in part-time roles or operating under contractual arrangements distinct from full-time employment were excluded from the study.

### 2.2 Study protocol

The study utilized paper-based questionnaires, which were delivered by the researchers to the hospital and subsequently disseminated to the intended participants by departmental nurses. Voluntary participation was emphasized throughout the study. Prior to commencement, all nurses were duly informed of the anonymous nature of the research and were assured of their prerogative to withdraw without the obligation of providing a rationale. They were instructed to complete the questionnaires at their convenience within a one-week timeframe and to return the finalized surveys in securely sealed envelopes. A total of one thousand questionnaire surveys were distributed across both hospital settings, yielding a response rate of 79.6% with 796 accurately completed questionnaires returned. The reasons underlying the non-participation of 204 respondents remain undisclosed. Demographic and occupational data were exclusively obtained from self-reported information provided within the surveys. The utilization of incentives to encourage study participation was not implemented.

### 2.3 Study group

The cohort under investigation comprised 796 nurses. A significant majority of the participants were female (94.1%). Nurses aged below 34 accounted for 35.2% of the sample, while those aged 51 and above represented 22.7% of the respondents. The mean age of the surveyed nurses was 41.5 years, with a standard deviation of 11.8 years. Furthermore, a notable 77.8% of the respondents had attained higher nursing education. Those with less than 6 years of work experience constituted 30.7% of the cohort, whereas individuals with over 17 years of experience comprised 39.7% of the participants.

### 2.4 Description of the questionnaire and the applied measures

The instrument employed for evaluating the health status was the Goldberg General Health Questionnaire GHQ-28, adapted for use in Poland by Makowska and Merecz (33). The GHQ-28 questionnaire serves as a tool for appraising the mental well-being of adult individuals, facilitating the identification of individuals whose mental states have been subject to temporary or enduring impairment due to life challenges, adversities, or mental disorders, as well as those at a significant risk of mental health complications. Apart from its overall scoring, the GHQ-28 questionnaire comprises four distinct scales, namely somatic symptoms, anxiety/insomnia, social dysfunction, and severe depression symptoms. The severity of these adverse mental states is evaluated by aggregating responses to specific queries, coded using a dichotomous system. The sample queries are: *have you had headaches recently?, have you been getting irritated and angry recently?, have you felt that what you are doing is useful recently?, have you felt that life is not worth living recently?.* The individual domain measures range from 0 to 7 points, while the total measures range from 0 to 28 points. Higher GHQ values are indicative of poorer mental health. The authors of the questionnaire have standardized these measures. Both the original GHQ-28 and its Polish adaptation have undergone extensive validation and are accompanied by clear scoring guidelines (34). The GHQ-28 questionnaire was also validated using data obtained from the study group, calculating the value of the Cronbach’s Alpha coefficient for the component measures and for the overall measure. The obtained results: somatic symptoms – 0.860, anxiety/insomnia – 0.893, social dysfunction – 0.848, severe depression – 0.890, total – 0.937, have very high values, confirming the desired psychometric properties of the GHQ-28 questionnaire.

The Subjective Work Evaluation Questionnaire (SWEQ) developed by Dudek and Waszkowska was administered to evaluate subjective work attributes (8). This tool serves to gage the subjective perception of work and is specifically designed to quantify employees’ personal experience of occupational stress. Comprising 50 statements delineating various job characteristics, each statement is accompanied by a numerical scale ranging from 1 to 5. The sample statements of the questionnaire are: *my job requires vigilance*, i.e., *readiness to react quickly to an important signal that may appear at any moment, working in my position, I do not have information about whether what I do is done well or poorly, in my work, I receive tasks that require me to compete with others, I sometimes come home with a sense of task non-completion.* This scale enables respondents to indicate the degree to which a particular attribute is perceived as burdensome. Notably, a rating of 1 implies the absence of the specific attribute within the job, while a rating of 5 signifies the highest level of perceived adversity. The questionnaire has undergone rigorous validation and is accompanied by clearly defined scoring protocols (8). The SWEQ questionnaire was also validated using results from the study group, calculating the value of the Cronbach’s Alpha coefficient for the overall measure and component measures. The obtained results (for the overall measure of general stress – 0.961, and for the specific measures ranging from 0.650 to 0.868) have very high values, indicating the desired psychometric properties of the SWEQ questionnaire.

### 2.5 Statistical methods

In the descriptive segment, we have presented an array of the community under examination, delineated in the form of tables incorporating numerical descriptors for quantitative attributes or percentage distributions for select characteristics.

We used the Mann–Whitney test to assess the significance of differences between the two groups, where one experienced a certain factor and the other did not. We analyzed the relationship between two numerical (ordinal) characteristics by determining the values of Spearman’s rank correlation coefficient (rS) and supplemented it with the results of the correlation coefficient significance test (*p*).

We used regression analysis to describe the impact of COVID-19 pandemic-related and psychosocial factors on all measures of workload in the SWEQ, and the overall measure of mental health assessment with the GHQ-28 questionnaire. Using the procedures of searching for the optimal model (stepwise regression, search for the best subset), we selected the factors that affect each measure in a statistically significant way. We then presented these models in the results and subjected them to interpretation.

## 3 Results

### 3.1 Subjective evaluation of negative work characteristics

The negative aspects of nurses’ work were subjectively assessed using the 50-item SWEQ questionnaire. This assessment served as the foundation for calculating score-based workload measures across 10 selected dimensions (6). These measures possess a pejorative nature, where higher values indicate a less favorable evaluation of work outcomes. Table 1 provides details on the distribution of numerical measures related to work characteristics.

**Table 1:** Subjective evaluation of negative work features.

Subjective work evaluation questionnaire	$\bar{x}$	Me	*s*	Min	Max	*A*
General stress	117.1	110	35.0	60	275	1.19
Work overload	19.3	18	6.8	–	45	0.96
Lack of rewards	16.3	15	6.7	8	40	0.92
Uncertainty in workplace	16.2	15	5.6	7	35	0.76
Social relations	10.6	–	3.3	5	25	1.65
Threat	11.9	11	3.8	5	25	0.75
Physical burdens	8.0	8	4.7	4	20	0.87
Unpleasant work conditions	5.2	3	3.2	3	15	1.32
Lack of control	8.7	8	2.7	4	20	1.74
Lack of support	5.4	5	2.4	3	15	1.18
Responsibility	9.2	–	3.0	4	20	0.86

The SWEQ questionnaire did not incorporate standardization measures from its authors, thus precluding direct comparisons. However, point thresholds were established for each questionnaire measure, and surpassing these thresholds signified the presence of adverse working conditions in a particular domain. Consequently, individuals falling into this category were identified as having a high level of negative work attributes according to the questionnaire’s creators’ criteria (6). Table 2 offers a summary of the figures and percentages of individuals exhibiting a high level of negative assessments pertaining to specific work aspects. The results, excluding overall stress, were ranked based on the frequency with which particular negative characteristics were reported, from the most commonly cited to the least frequently mentioned by r**e**spondents.

**Table 2:** Number and percentage of people with high levels of negative work characteristics.

Subjective work evaluation (negative ratings)	Count	Percentage^1^
Social relations	619	77.8%
Threat	562	70.6%
Responsibility	560	70.4%
Work overload	500	62.8%
Lack of control	472	59.3%
Lack of rewards	470	59.0%
Uncertainty in workplace	448	56.3%
Lack of support	445	55.9%
Physical burdens	408	51.3%
Unpleasant working conditions	316	39.7%
General stress	500	62.8%
No negative ratings	29	3.6%

1 The total does not have to be 100% because the test subjects could indicate any number of response options.

### 3.2 General mental health of the nurses

The numerical characteristics of the GHQ-28 measures are shown in Table 3. Each measure takes values from 0 to 7 points, while the summary measure takes values from 0 to 28 points. Comparing the mean values with the median, it can be seen that all measures have a very asymmetric distribution – the means are much higher than the median, which is 0 for the two measures more related to mental aspects, meaning that most of the people surveyed do not show any negative symptoms in these areas. According to our results, the GHQ-28 summary measure alone has slightly less asymmetry. Such a significant asymmetry markedly limits the ability to analyze detailed GHQ measures, as they simply show little variability.

**Table 3:** General mental health of the nurses.

GHQ-28	$\bar{x}$	Me	*s*	Min	Max	*A*
Somatic symptoms	2.05	1	2.20	0	7	0.84
Anxiety/insomnia	1.87	1	2.23	0	7	0.96
Social dysfunction	1.12	0	1.83	0	7	1.80
Severe depression	0.38	0	1.13	0	7	3.74
Total	**5.42**	**3**	**6.21**	**0**	**28**	**1.34**

### 3.3 The COVID-19 pandemic and psychometric measures

We analyzed the impact of selected factors resulting from the ongoing COVID-19 pandemic on individual measures of subjective evaluation of working conditions (SWEQ) and nurses’ self-assessment of health (GHQ-28) in detail. We used the Mann–Whitney test to assess the significance of differences between groups declaring contact with a given pandemic agent or lack thereof.

#### 3.3.1 Subjective evaluation of nurses’ work during the COVID-19 pandemic period

##### 3.3.1.1 Contact with a patient diagnosed with SARS-CoV-2 virus infection

We found no major differences in the assessment of psychosocial burden at work according to contact with a patient diagnosed with SARS-CoV-2 virus infection. The sense of no control was the only aspect significantly higher in those who have had contact with a patient diagnosed with SARS-CoV-2 virus infection than in those who have not had such contact.

##### 3.3.1.2 Home isolation

We found no major differences in the assessment of psychosocial burdens at work depending on whether home isolation was implemented or not. According to the outcome of the survey, home isolation only slightly differentiates feelings of responsibility (negative feelings in this aspect are higher among nurses who have been in home isolation).

##### 3.3.1.3 Staying in quarantine

Staying in quarantine does not differentiate any of the measures for assessing working conditions.

##### 3.3.1.4 SARS-CoV-2 test performed

Performing the SARS-CoV-2 virus test was the only pandemic factor that differentiated the group of nurses testing for COVID-19 from the non-testing group, which constituted a much smaller group. Those testing experienced higher levels of general stress, uncertainty in the workplace, threat and discomfort in the area of responsibility. The difference in the category of unpleasant working conditions is also on the verge of statistical significance. Detailed data are shown in Table 4.

**Table 4:** Undergoing SARS-CoV-2 virus testing vs. subjective evaluation of job characteristics.

Subjective evaluation of work	SARS-CoV-2 test performed						*p*
	Yes (*N* = 691)			No (*N* = 105)			
	$\bar{x}$	Me	*s*	$\bar{x}$	Me	*s*	
General stress	118.0	110	35.3	111.1	103	32.7	0.0410*
Work overload	19.4	18	6.9	18.5	18	6.5	0.3008
Lack of rewards	16.4	15	6.7	15.5	14	6.7	0.1358
Uncertainty in workplace	16.4	16	5.6	14.9	14	5.3	0.0048**
Social relations	10.6	-	3.3	10.3	-	3.3	0.4142
Threat	12.0	11	3.8	11.2	11	3.6	0.0391*
Physical burdens	8.1	8	4.8	7.4	8	4.0	0.3139
Unpleasant working conditions	5.3	3	3.2	4.7	3	3.0	0.0649
Lack of control	8.7	8	2.8	8.7	8	2.6	0.4580
Lack of support	5.4	5	2.4	5.2	5	2.2	0.6186
Responsibility	9.3	-	3.0	8.7	8	3.1	0.0121*

##### 3.3.1.5 COVID-19 virus infection

We found no major differences in the assessment of psychosocial burden depending on the history of SARS-CoV-2 virus infection. Only COVID-19 survivors had a greater sense of discomfort at work in the categories of social relations and responsibility.

#### 3.3.2 Mental health assessment of nurses during COVID-19 pandemic period

##### 3.3.2.1 Contact with a patient diagnosed with SARS-CoV-2 virus infection

We found no major differences in nurses’ assessments of mental health according to contact with patients diagnosed with SARS-CoV-2 virus infection. Only nurses who had contact with a patient diagnosed with SARS-CoV-2 virus infection had more severe somatic symptoms (statistically significant difference) than those who had no such contact.

##### 3.3.2.2 Home isolation

Being subject to home isolation did not affect the nurses’ self-assessment of mental health.

##### 3.3.2.3 Staying in quarantine

Being in quarantine did not affect the nurses’ self-assessment of their mental health.

##### 3.3.2.4 SARS-CoV-2 test performed

Nurses who were tested for SARS-CoV-2 have much stronger levels of anxiety and insomnia, and also have significantly stronger somatic symptoms. The overall health score is also significantly worse in this group. Detailed data are shown in Table 5.

**Table 5:** Undergoing SARS-CoV-2 virus testing vs. self-rated mental health.

GHQ-28	SARS-CoV-2 test performed						*p*
	Yes (*N* = 691)			No (*N* = 105)			
	$\bar{x}$	Me	*s*	$\bar{x}$	Me	*s*	
Somatic symptoms	2.10	1	2.20	1.69	1	2.20	0.0334*
Anxiety/insomnia	1.94	1	2.25	1.40	0	2.04	0.0056**
Social dysfunction	1.14	0	1.84	0.99	0	1.76	0.5272
Severe depression	0.39	0	1.15	0.29	0	1.01	0.3676
Total score	5.58	3	6.25	4.36	2	5.90	0.0226*

##### 3.3.2.5 SARS-CoV-2 virus infection

We found no major differences in mental health scores depending on the history of SARS-CoV-2 virus infection. Only the past SARS-CoV-2 infection is associated with slightly stronger symptoms of depression, although the difference is not very statistically pronounced.

### 3.4 Multivariate analysis

#### 3.4.1 Factors affecting subjective measures of stress at work

Using regression analysis, we constructed models to describe the effects of socio-professional factors (age, years of work, education, work system) and SARS-CoV-2-related factors on all subjective measures of work stress. Employing the procedures of searching for the optimal model (stepwise regression, search for the best subset), we then selected those factors that affect each measure in a statistically significant way.

Among the socio-occupational factors, only years of work and 12-h (vs. 8 h) work system resulted in experiencing greater general stress, work overload, lack of rewards, uncertainty in workplace, social relations and feeling of threat. For factors related to the pandemic, correlations occurred only for being tested for SARS-CoV-2, being subject to home isolation and being infected with SARS-CoV-2. Those who were tested for SARS-CoV-2 had a higher sense of uncertainty in workplace, by 1,422 points on average (Table 6). SARS-CoV-2 virus infection increased negative feelings about social relations at work, while staying in social isolation decreased them (Table 7). Those who had SARS-CoV-2 infection had, 0.575 points higher feelings of threat at work on average than those who did not have the infection (Table 8).

**Table 6:** The impact of socio-occupational factors and those resulting from the COVID-19 pandemic on perceptions of uncertainty in workplace.

Independent factors	Uncertainty in workplace *R*^2^ = 2.2% *F* = 5.9 *p* = 0.0005***		
	*B* (95% c.i.)	*p*	*ß*
SARS-CoV-2 test	1.422 (0.268; 2.576)	0.0158*	0.09
Years of work	0.051 (0.010; 0.092)	0.0158*	0.09
Work system (12 vs. 8 h)	0.866 (−0.028; 1.761)	0.0577	0.07

**Table 7:** The impact of socio-occupational factors and those resulting from the COVID-19 pandemic on social relations.

Independent factors	Social relations *R*^2^ = 3.2% *F* = 6.3 *p* = 0.0001***		
	*B* (95% c.i.)	*p*	*ß*
Staying in home isolation	−0.506 (−1.037; 0.026)	0.0621	−0.08
SARS-CoV-2 virus infection	0.694 (0.165; 1.223)	0.0102*	0.11
Years of work	0.036 (0.012; 0.060)	0.0035**	0.10
Work system (12 vs. 8 h)	0.735 (0.216; 1.254)	0.0056**	0.10

**Table 8:** The impact of socio-occupational factors and those resulting from the COVID-19 pandemic on feelings of threat.

Independent factors	Threat *R*^2^ = 2.6% *F* = 5.2 *p* = 0.0004***		
	*B* (95% c.i.)	*p*	*ß*
SARS-CoV-2 virus infection	0.574 (0.040; 1.107)	0.0350*	0.07
Age	−0.061 (−0.105; −0.017)	0.0063**	−0.19
Years of work	0.087 (0.032; 0.142)	0.0020**	0.21
Work system (12 vs. 8 h)	0.683 (0.067; 1.298)	0.0298*	0.08

#### 3.4.2 Factors affecting the GHQ-28 total health score

Using regression analysis, a model was developed to describe the impact on the overall measure of mental health according to the GHQ-28 of socio-professional factors (age, years of work, education, work system) and factors related to the COVID-19 pandemic. Detailed measures from the GHQ-28 questionnaire were not subjected to regression analysis because they show excessively homogeneous distribution – most values are 0.

The model for the overall ill-being symptom score does little to explain the variation of this characteristic – the coefficient of determination is only 2.5%. The years of work trait was most strongly associated with the overall mental health score. Subjects who tested positive for SARS-CoV-2 had worse general well-being. The GHQ-28 overall measure is higher, by about 1,282 points on average (Table 9).

**Table 9:** The impact of socio-occupational factors and those resulting from the COVID-19 pandemic on the overall mental health assessment score.

Independent factors	General results *R*^2^ = 2.5% *F* = 6.5 *p* = 0.0002***		
	*B* (95% c.i.)	*p*	*ß*
SARS-CoV-2 test	1.282 (0.001; 2.563)	0.0499*	0.07
Years of work	0.085 (0.039; 0.131)	0.0003***	0.13

## 4 Discussion

The nurses surveyed rated their work as highly taxing. Nursing’s high position in rankings of the most stressful professions confirms the validity of such an assessment (34, 35). In our survey, a substantial 62.8% of respondents categorized the perceived overall level of work-induced stress as very high. Some components of the comprehensive SWEQ questionnaire were rated as very high by an even larger proportion of participants. Social relations induced very high stress in 77.8% of the respondents, in 70.6% - by threat, and in 70.4% – by responsibility. Only unpleasant work conditions, indicated as a highly stressful factor by only 39.7% of respondents, significantly differed from the other measures. The high percentage of scores considered high may be due to the fact that the authors of the questionnaire set normal ranges by taking different occupations into account (8). This implies that nurses tend to identify a relatively higher number of negative aspects in their work in comparison to other occupations. All measures of the SWEQ questionnaire were characterized by right-handed asymmetry, meaning that more nurses surveyed described the presence of burdens at a low or average level than at a high level. Similar results were obtained in studies conducted in hospitals in other parts of the country (36, 37). In surveys of nurses from other countries, work overload was the most stressful aspect of the job. In terms of other stressful traits occurring, we have not been able to identify a significant pattern among published research findings (38–40), which is likely due to cultural differences and the use of different research tools.

Overall, the nurses rated their mental health fairly well. Most subjects did not indicate symptoms of social dysfunction or severe depression. However, the distribution of GHQ-28 mental health measures in our studies is characterized by high asymmetry. The values of the GHQ-28 measures had right-sided asymmetric distributions, in particular, a very high asymmetry index appeared for symptoms of depression. This means that for most people, the level of health complaints was low (and, indeed, the median score was 0 for dysfunction and depression, meaning at least half of the respondents had no complaints in terms of this aspect). Somatic symptoms were the most frequent, while severe depression was the least frequent. With regard to this, our results differ from that obtained in a study conducted in Iran (41), where the most common disorder was social dysfunction by far, with somatic symptoms only in third place. Moreover, according to Greek researchers, depressive conditions were far more common than in our country, and anxiety/insomnia were reported on a similar level (42). Studies conducted in other regions of Poland and Lithuania reported results similar to ours (43, 44).

The COVID-19 pandemic has had a huge impact on the lives of entire societies, and in particular on the operation of health services. The issue has been extensively studied, and there has been a negative impact in most cases (29, 30, 35). Still, upon comparing the present results to our results conducted before the COVID-19 pandemic was announced, we found no significant differences in the subjective assessment of job characteristics (SWEQ) or the nurses’ self-assessment of mental health (GHQ-28). Both studies were conducted on a similar group of nurses working in hospitals in the Podlaskie region (45).

In the subsequent step of the analysis, we compared how selected pandemic-related elements, i.e., direct contact with a patient infected with SARS-CoV-2, staying in quarantine, home isolation, being tested for the virus and being infected with SARS-Cov-2 itself, are correlated with subjective assessment of workplace burdens and self-assessment of mental health in groups of nurses who were directly exposed to these factors or not. Overall, it can be said that the differences between the groups are small. For the subjective assessment of work characteristics, we found no correlation of most pandemic factors with the occurrence of individual workloads. Undergoing virus testing was the only factor worth noting. Nurses tested experienced higher levels of general stress, uncertainty in the workplace, a sense of threat and discomfort in the area of feeling responsible.

Being tested for the virus was also the only factor that differentiated the groups in terms of self-rated mental health. Nurses subjected to Sars-CoV-2 virus testing demonstrated a 38.6% heightened intensity in anxiety and insomnia, alongside a 24.2% increased severity in somatic symptoms compared to their non-tested counterparts. Moreover, the overall GHQ-28 score among tested nurses registered a 30.0% reduction compared to those who did not undergo testing. It can be concluded that testing had a poor effect on nurses’ working conditions, as it introduced additional nervousness. Genetic tests were the ones performed during the study period, and their result was known only after several or over a dozen hours. One can safely surmise that the anticipation of the test result and the accompanying fear of being infected, was the most stressful factor, rather than the test itself. Similar results were obtained by researchers in China (30), where nurses were also most concerned about whether they were infected and whether they could transmit the virus to others. Other researchers confirm the effect of factors related to the COVID-19 pandemic as stressors, but also fail to show their direct effect on subjective feelings of stress and mental health of health workers (46, 47).

Using regression models, we studied how demographic and occupational factors, as well as factors related to the COVID-19 pandemic, might affect the subjective assessment of job characteristics according to the SWEQ questionnaire, and nurses’ mental health assessment with the GHQ-28 summary measure. Amid the factors linked to the COVID-19 pandemic, the process of undergoing SARS-CoV-2 virus testing was linked to a 1.422-point increase in feelings of uncertainty within the workplace, accompanied by a 1.282-point decline in self-rated mental health. This confirms the results obtained in the correlation analysis and is consistent with the results reported by other researchers, according to which intensive testing of nurses a significantly stressful factor is (48–50). This raises the question: has intensive testing of nurses, especially asymptomatic nurses, done more harm to the mental health of workers than good, in terms of controlling the pandemic spread? Perhaps it would be worthwhile to order testing with greater caution in general in the case of future outbreaks.

Virus infection yielded a 0.574-point augmentation in perceived threat and a 0.694-point reduction in social interaction. The interpretation of this result seems fairly obvious (51). An infected individual usually feels a threat to their health or even life, and as an emitter of the “pestilence,” experiences stress about whether they have spread the virus to their loved ones or co-workers, or experiences grudge with regard to those who potentially infected them, especially during the COVID-19 pandemic, where the media constantly broadcast information spreading the fear of havoc wrought by the virus.

Two factors associated with the COVID-19 pandemic surprisingly reduced some of the psychosocial burden on nurses in the workplace. Home isolation was associated with a 0.506-point enhancement in social interaction. Paradoxically, in this day and age, when a significant portion of human contact has been digitized through the proliferation of social media and video platforms, being in seclusion allows for intensification of social contacts (52). Interactions with an infected patient were linked to a 0.499-point decrease in work-related discomfort attributed to a perceived lack of control. This phenomenon is difficult to explain logically.

We did not find any effect of age and education level on the evaluation of work characteristics or mental health. There were relationships found with regard to years of work experience and the work system. Employees with greater tenure demonstrated heightened perceptions of lack of rewards, increased workplace uncertainty, altered social relations, heightened sense of threat, lack of support, heightened responsibility, and elevated general stress levels. Additionally, nurses on 12-h shifts reported amplified psychological strain, increased perception of insufficient rewards, altered social relations, heightened sense of threat, heightened physical burdens, unpleased working conditions, lack of control, elevated sense of responsibility, and increased general stress compared to their counterparts on 8-h shifts. Notably, in the context of self-assessed mental health, we observed a statistically significant relationship solely between extended work experience and poorer health ratings. The findings obtained appear to align closely with established trends documented in previous research (25, 30). Working on a 12-h system is more taxing in all respects, both physically and mentally. In the case of seniority, some studies confirm greater resilience to workplace stress, and others (as in our case) just the opposite (53, 54). The low statistical strength of the obtained correlations is noteworthy, not only in terms of our study (55). This shows that the feeling of strain at the workplace is a very complex process that depends on a great number of factors in an individualized way, and also depends on numerous conditions that are extremely difficult to list.

The COVID-19 pandemic’s impact on healthcare services has been widely studied (56, 57), yet our recent analysis, compared with pre-pandemic data, revealed no significant changes in nurses’ perceptions of job characteristics or self-assessed mental health. Notably, virus testing emerged as a significant stressor, associated with heightened stress levels and feelings of organizational threat. Infection was linked to increased perceived threat and decreased social interaction, reflecting the psychological strain and fear amid media coverage. Counterintuitively, home isolation exhibited a marginal enhancement in social relationships, while contact with infected patients yielded a slight alleviation of discomfort related to the perceived lack of control, which was not reflected in the results of other researchers. These findings underscore the complexities of pandemic-related stressors and highlight the need for targeted interventions and support strategies for healthcare workers in future crises.

## 5 Conclusion

1. The COVID-19 pandemic did not change nurses’ subjective perceptions of job characteristics, nor did it change their self-assessment of mental health.
2. The impact of factors stemming from the COVID-19 pandemic, including exposure to infected patients, quarantine or home isolation, and even SARS-CoV-2 virus infection, did not significantly influence nurses’ self-assessment of their mental well-being.
3. In the event of future pandemics, managers should reconsider the need to intensively test nurses for the SARS-CoV-2 virus, as testing has been a factor in intensifying nurses’ feelings of negativity about their work and worsening their self-assessment of their mental health.

## 6 Methodological limitations

The utilization of a limited sample, adoption of a cross-sectional study design, and reliance on self-reported questionnaires represent noteworthy constraints of this investigation. Notably, the study was confined to two hospitals situated within a single region of Poland. Furthermore, the specific reasons underlying the non-participation of 30.4% of the invited individuals remain undetermined, primarily attributable to the procedural nature of the study. It is essential to emphasize that prior and contemporaneous studies involving analogous cohorts of nurses were executed, albeit not among identical participant groups, both preceding and during the COVID-19 pandemic.

## Data availability statement

The raw data supporting the conclusions of this article will be made available by the authors, without undue reservation.

## Ethics statement

The studies involving humans were approved by the Bioethical Committee. Medical University of Bialystok, Bialystok, Poland. The studies were conducted in accordance with the local legislation and institutional requirements. The participants provided their written informed consent to participate in this study.

## Author contributions

KK: Conceptualization, Investigation, Methodology, Supervision, Writing – original draft, Writing – review & editing. KT: Conceptualization, Validation, Writing – review & editing. JC: Investigation, Validation, Writing – review & editing. EK-K: Methodology, Writing – review & editing. MS: Data curation, Software, Writing – original draft, Writing – review & editing. JH: Formal analysis, Methodology, Writing – review & editing.
